# Context and Time Matter: Effects of Emotion and Motivation on Episodic Memory Overtime

**DOI:** 10.1155/2018/7051925

**Published:** 2018-04-08

**Authors:** Qing Sun, Simeng Gu, Jiongjiong Yang

**Affiliations:** ^1^School of Psychological and Cognitive Sciences and Beijing Key Laboratory of Behavior and Mental Health, Peking University, Beijing 100080, China; ^2^Department of Medical Psychology, Jiangsu University Medical School, Zhenjiang 212013, China

## Abstract

Previous studies have shown that compared with neutral cues, stimuli with positive and negative/stressful contexts or reward and punishment cues are remembered better. However, it is unclear whether the enhanced effect differs in emotion or motivation dimensions and the passage of time. We addressed these issues by manipulating different contextual cues for neutral words at different time intervals. In experiment 1, subjects were asked to learn words with picture contexts in positive, negative/stressful, and neutral valences and were tested by old/new word recognition and contextual judgment 10 min, 1 day, and 1 week later. In experiment 2, the reward and punishment motivations were manipulated by monetary cues during learning. Word recognition and contextual judgment were assessed 10 min, 1 day, 1 week, and 1 month after the study. Compared with negative and punishment conditions, the words in positive and reward contexts were recognized better at shorter intervals, which was associated with recollection process. In contrast, the words in negative and punishment contexts were recognized better at longer intervals, which was mainly associated with familiarity process. These results clarified how different dimensions of emotional and motivational contexts influence memory at short and long intervals and highlighted the role of contextual features in memory formation and consolidation.

## 1. Introduction

We encounter enormous information every day, but only a small portion of the information is remembered and remained in the long-term memory. In recent years, studies have suggested that in addition to some salient events (e.g., emotional faces and stressful events), emotional and motivational contexts can also enhance memory for associated neutral events. The negative/stressful and positive stimuli are usually used as emotional contexts [1–4] and monetary reward and punishment/loss as motivational contexts [5, 6]. The electronical shock is used as punishment [7, 8] or negative manipulation [9].

Emotional and motivational contexts influence memory when they are presented in different phases. The contexts can be presented before/with the stimuli (as cues), or after the stimuli, leading to proactive or retroactive memory enhancement. For example, memory for face names was enhanced when happy expressions were presented as cues [10], and memory for neutral pictures was enhanced when negative cues were used in 5 min delay [11]. With regard to motivational contexts, in a study by Adcock et al. [5], participants were presented with pictures that were labeled with high- and low-reward cues. The recognition performance was better in the high- versus low-reward condition 24 h later. The neutral words with shock cues were recognized better than those without shock cues 24 h later [7]. In addition, the enhancement for reward contexts is associated with a high confidence [5] and recollection process [12, 13]. In a study of Gruber et al. [12], subjects learned the object-scene associations in high- or low-reward condition and tested them with object and object-scene associations about 30 min later. Compared with the low-reward condition, the associations that were learned in the high-reward condition were remembered better and relied on the recollection contribution.

One interesting question addressed in this study is whether the memory enhancement by emotional or motivational contexts is time-dependent. As memory is generally forgotten with the passage of time [14], it is unclear whether the enhancements remain for a long time. Previous studies have suggested that emotional and motivational contexts influence both encoding and consolidation stages. Emotional and motivational stimuli could attract more attention during encoding [15–17]. In addition, negative/stressful stimuli are usually highly arousing, which triggers a more efficient consolidation by the interaction of the amygdala and hippocampus system [18]. Motivational stimuli, on the other hand, enhance memory consolidation by the interaction of dopamine and hippocampus system [19]. The studies using the retroactive memory paradigms provided additional evidence for the consolidation mechanisms. For example, Murayama and colleagues showed that monetary reward cues enhanced proactive memory for irrelevant objects, but the effect was observed only after 24 h, not immediately upon testing [20, 21].

Although it is possible that emotional and motivational contexts lead to stable memory enhancement over time, current findings are not clear. Most studies found the enhanced memory for emotional contexts immediately after learning [1–3, 11]. Some studies found the enhanced effect at 24 h but not at short intervals [9, 22]. For the motivational manipulation, some studies found the reward-related memory enhancement for neutral pictures shortly after study (1 min in Shigemune et al.; 30 min in Murty et al.) [8, 23] or 24 h later [5, 7]. But others showed the reward-related enhancement in 3 weeks but not immediately after study [6]. Few studies compared the memory enhancement between different time intervals longer than 1 week [6].

The inconsistent findings may be due to the fact that memory enhancement differs in types of contexts with the passage of time. When memories with different contexts were directly compared, positive contexts enhanced memory more strongly than negative (and neutral) cues for target pictures [2, 3], words [1], and faces [24] at the day of encoding. Other studies using actual shocks during encoding found the enhanced effect at 24 h but not at short intervals [9, 22]. With regard to motivational contexts, a study by Murty et al. [8] compared the effects of monetary reward and mild shock on subsequent recognition of surprise events 30 min after they were learned. The results showed that events with reward motivation resulted in higher memory performance than those with shocks. Note that the memories in positive/reward contexts were enhanced minutes or 1 day after the encoding, which raised the possibility that memory enhancement for the positive and reward contexts occurs earlier, whereas that for the negative contexts occurs later in memory stage. But studies of different emotional and motivational contexts varied in the testing intervals, most only after short delays [8, 23]. We know little about how the memory enhancement changes over time, and it is necessary to include both the time interval and different context as independent factors to address this issue.

If memory enhancement for different contexts differs in time intervals, we would find different contribution of recollection and familiarity processes over time. On the one hand, the memories for positive and reward and the memories for negative and punishment contexts depend on recollection and familiarity differently. For example, the reward-related memory is associated with recollection [6, 12]. The enhanced memory for neutral scenes associated with shock is associated with the familiarity rather than the recollection [9]. On the other hand, previous studies have suggested that memory relying on recollection and familiarity processes differs in forgetting characteristics [25]. As stated in Sadeh et al. [25], memories relying on recollection are forgotten primarily due to decay over time but are relatively resistant to interference from irrelevant information. By contrast, memories relying on familiarity are prone to interference but show less effect of decay. Therefore, when memory enhancement due to different emotional contexts and motivations is associated with recollection and familiarity processes, the forgetting rate would differ over time.

Combining the study for memory with emotional and motivational contexts is important, because both of the contexts are commonly used in memory studies, but it is unclear whether they have similar effects on memory enhancement and whether they rely on similar mechanisms. Some studies showed that both electronical shock and money loss led to similar activation in the striatum [26], and positive emotion evoked value representations in the striatum [27]. In addition, facilitation of memory by punishment motivation may recruit similar neural circuitry as threatening items in the amygdala [7] and medial temporal regions [9]. Clarifying their relationship in behavioral level would provide insights on studies on neural mechanisms.

In sum, the objective of the study was to explore to what extent different emotional and motivational contexts influence memory for neutral targets over time. We addressed these issues by manipulating different contextual cues for neutral words at different time intervals. In experiment 1, pictures with positive, negative/stressful, and neutral valences were used as contexts of words [2, 3, 28]. To dissociate the effects of valence and arousal, the arousal levels of positive and negative/stressful pictures were matched. After subjects learned the associations between words and contexts for 10 min, 1 day, and 1 week, they were tested by old and new word recognition and contextual judgment. We did not include 1-month interval in experiment 1, because that the memory performance was at chance level at 1 month in pilot studies. In experiment 2, the reward and punishment motivation was manipulated by monetary cues during learning and subjects' fees afterward. The motivational levels of punishment and reward were also matched by a separate rating, which was also confirmed by participants' post hoc reports. The word recognition, remember/know judgment, and contextual judgment were performed 10 min, 1 day, 1 week, and 1 month after the study. Based on previous studies, we hypothesized that emotional and motivational contexts enhance memory for subsequent words. In comparisons of different contexts, memory in positive and reward conditions depends on recollection and is higher than that in negative and punishment conditions at shorter intervals, whereas memory enhancement for negative and punishment pictures may be more familiarity-based and could last for a longer time.

## 2. Materials and Methods

### 2.1. Experiment 1

In experiment 1, by using the emotional contextual paradigm, we explored whether negative or positive contexts enhanced memory for neutral words differently over time. The negative and positive pictures were matched in their arousal levels. Participants were first presented with neutral words, followed by pictures of positive, negative, and neutral valences overlaid on the neutral words. They were tested for memories of words and contextual information after 10 min, 1-day, and 1-week intervals.

#### 2.1.1. Participants

Twenty-eight healthy, right-handed participants (10 males) with a mean age of 22.1 ± 2.2 years were recruited in the study. All of the participants were native Chinese speakers, and they all provided written informed consent in accordance with the procedures and protocols approved by the Review Board of School of Psychological and Cognitive Sciences, Peking University.

#### 2.1.2. Material

**Figure 1:**
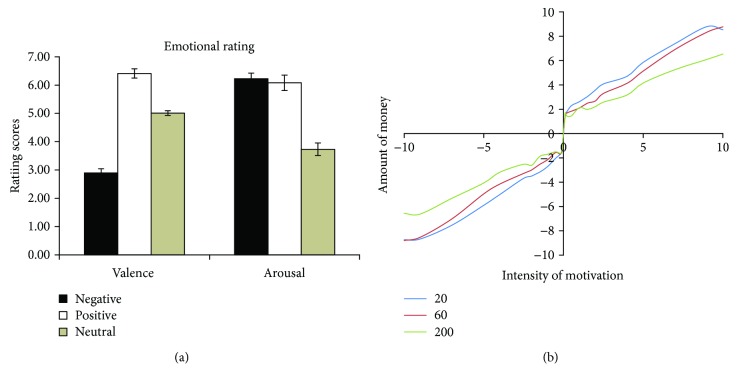
Emotional and motivational rating in experiments 1 and 2.

Two within-subject factors were included in the study: context (positive, negative, and neutral) and time interval (10 min, 1 day, and 1 week). The contexts were 180 pictures, of which negative, neutral, and positive pictures each having 60 pictures. Another 18 participants (11 males, average age 23 ± 1.97 years) rated the pictures in the dimensions of valence and arousal. The three types of contexts differed significantly in valence (2.90 ± 0.61 for negative, 6.41 ± 0.69 for positive, and 5.01 ± 0.36 for neutral, *F*(2,34) = 157.90, *P* < 0.001, *η*^2^ = 0.90) and arousal (6.23 ± 0.83 for negative, 6.08 ± 1.15 for positive, and 3.73 ± 0.95 for neutral, *F*(2,34) = 114.21, *P* < 0.001, *η*^2^ = 0.87) ratings (Figure 1(a)). Negative pictures had the lowest valence rating scores (*P*′s < 0.001). More important, the negative and positive pictures were comparable in arousal rating (*P* = 0.99), and both were rated higher in arousal than the neutral pictures (*P*′s < 0.001). Thus, the arousal level was optimally controlled in the experiment.

The neutral words were 360 Chinese nouns, half of which were abstract (e.g., courage) and the other half were concrete (e.g., bedding). They had middle level of word frequency (26.56 ± 92.99) and number of strokes (17.30 ± 4.93). The words were divided into two sets, one set as learning materials and the other as new words during retrieval. Each set was further divided into three subsets. The subsets were matched in frequency and number of strokes (*F*'s < 1). The pictures were divided into four sets. Each set had 20 negative pictures, 20 neutral pictures, and 20 positive pictures. The four sets were used as the contexts for four time intervals. The four sets were matched in their valence and arousal ratings (*F*'s < 1). The 180 pictures and 180 words were formed into 180 pairs that did not have close semantic relationship to each other. The materials were counterbalanced so that each picture-word pair had an equal chance to be the material for each condition.

#### 2.1.3. Procedure

**Figure 2:**
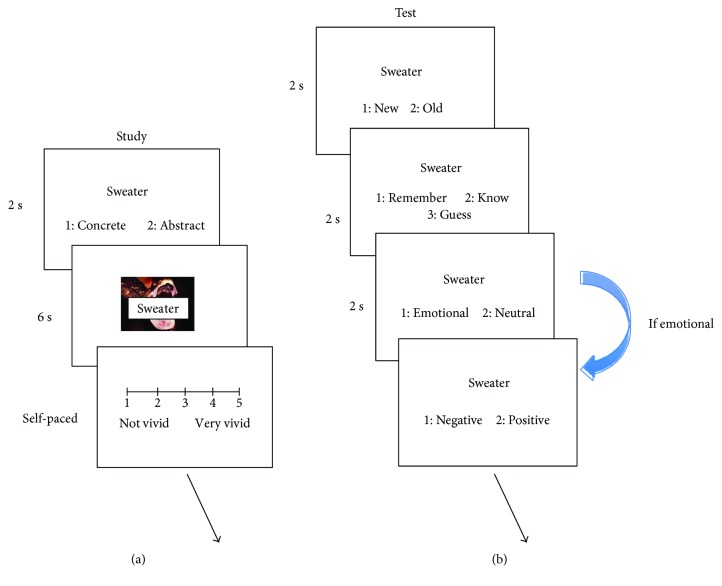
Procedure of the study and test sessions in experiment 1. During encoding, participants first performed a concreteness judgment for each word and then performed an imagination task to combine the picture and the word. During retrieval, the participants made word recognition, R/K/G judgment, and source judgment for each word. Chinese words are replaced by English words for illustration purpose.

During the study phase (Figure 2(a)), a neutral word was first presented for 2 s for each trial, and the participants were asked to judge whether the word was a concrete noun or an abstract noun. Then the combination of picture and word was presented for 6 s, during which participants were asked to remember the word and its link with the picture to imagine a scene, followed by the task of making a subjective evaluation of the vividness of the imagination they made (1 refers to not vivid at all, and 5 refers to extremely vivid). All stimuli were pseudorandomly presented during the encoding phase so that no more than three stimuli that were tested in the same time interval and with the same valence were presented consecutively.

During the test phase, a word was presented on the screen for 2 s for each trial, and the participants judged whether the word was old or new as accurately and quickly as possible (Figure 2(b)). If the word was judged as “old,” the word was presented for 1 s again, and the participants were asked to make remember/know/guess judgment. The response with “remember” was made when the participants could retrieve stimulus-related details or contexts; the response with “know” was made when they only felt that the stimulus was familiar without any detailed information. The response of “guess” was made when they retrieved the stimulus by the previously mentioned two processes, they responded with “guess.” Finally, the word was presented again, and the participants judged whether the context picture was emotional/neutral or negative/positive. The old and new words were pseudorandomly presented for each time interval, so that no more than three words that were in the same valence were presented consecutively.

The participants learned the 180 words in the same day and then performed the recognition tests at three time intervals (with different words). Before each test phase, a 5 min distraction task was performed to avoid a rehearsal from the study phase (i.e., count backward by 7 continuously from 1000). The participants had separate opportunities to practice the study and test trials before the formal phase.

#### 2.1.4. Data Analysis

The hit rate (Hit), false alarm rate (FA), corrected recognition (Hit-FA), and the mean reaction times (RTs) were calculated and analyzed separately, using a repeated measures ANOVA with the time interval (10 min, 1 day, and 1 week) and the emotional contextual type (positive, negative, and neutral) as within-subject factors. Four subjects' data were excluded due to lower hit rates (>2 SD) at 10 min. The remaining 24 subjects' results were used for data analysis. The *d*′ was also calculated for each subject and averaged according to signal detection theory. Because the results of the *d*′ value and corrected recognition were similar, only the corrected recognition results were reported in detail. The RTs were based on the mean RTs and only correct responses were included in the analysis. The forgetting rate was estimated by the interaction between the retention interval and the memory type [29, 30]. Partial eta squared (*η*^2^) was calculated to estimate the effect size of each analysis. Post hoc pairwise comparisons were Bonferroni-corrected (*P* < 0.05, two-tailed).

Recollection and familiarity processes were estimated using the independent K (IRK) procedure [31, 32], in which *R* responses were assumed to estimate recollection, whereas familiarity was estimated as the proportion of *K* responses divided by the proportion of non-*R* responses. By this, the *R* and *K* responses were not only mutually exclusive but also independently estimated. Then *R* and IRK responses were corrected using the FA: recollection = *p*(*R*, Hit)−*p*(*R*, FA); familiarity = *p*(*K*, Hit)/(1−*p*(*R*, Hit))−*p*(*K*, FA)/(1−*p*(*R*, FA)) [31–33]. Repeated measures ANOVA with the time interval (10 min, 1 day, and 1 week) and the emotional contextual type (negative, positive, and neutral) as within-subject factors was performed.

### 2.2. Experiment 2

In experiment 2, we explored whether punishment or reward motivation modulated memory differently over time. Participants learned the same neutral words as those in experiment 1. In one-third of the trials, they were told that if they remembered the word during the test, they would be rewarded afterward. In another third of the trials, if they did not remember the word afterward, they would be punished. In the last third of the trials, if they remembered the words, they were neither rewarded nor punished. The motivational intensity was assessed and matched for reward and punishment conditions to exclude its potential effect on memory performance [34]. Word recognition and source memory were tested after different time intervals.

#### 2.2.1. Participants

Twenty-eight healthy, right-handed participants (12 males) with a mean age of 22.0 ± 2.64 years were recruited in the study. All of the participants were native Chinese speakers, and they all provided written informed consent in accordance with the procedures and protocols approved by the Review Board of School of Psychological and Cognitive Sciences, Peking University.

#### 2.2.2. Materials

Two within-subject factors were included in the study: motivational context (reward, punishment, and control) and retention interval (10 min, 1 day, 1 week, and 1 month). The words were the same as those in experiment 1. The motivation was manipulated with symbols. The symbol “↑ 6 yuan” referred to the reward condition, symbol “↓ 6 yuan” referred to the punishment condition, and “0 yuan” referred to the control condition.

Before the formal experiment, another 10 participants (5 males, 23 ± 1.62 years old) rated the motivational intensity of the punishment and reward conditions. The participants were asked to rate their motivation to remember the word when they would get the reward for remembering or when they would get the punishment for forgetting. We asked the participants to rate their motivational intensity from 1 to 9 (lowest to highest). The amount of money varied from 0.1 yuan to 0.2 yuan, 0.5 yuan, 1 yuan, 5 yuan, and 10 yuan. To control the influence of the total amount of money on the rating, the participants were told that they would pay 20 yuan, 60 yuan, and 200 yuan, respectively, after the test. The results showed that the motivational intensity increased linearly when the reward or punishment levels increased (Figure 1(b)), irrespective of the total test fee. Thus, we chose 6 yuan as the level of punishment and reward in the experiment.

#### 2.2.3. Procedure

**Figure 3:**
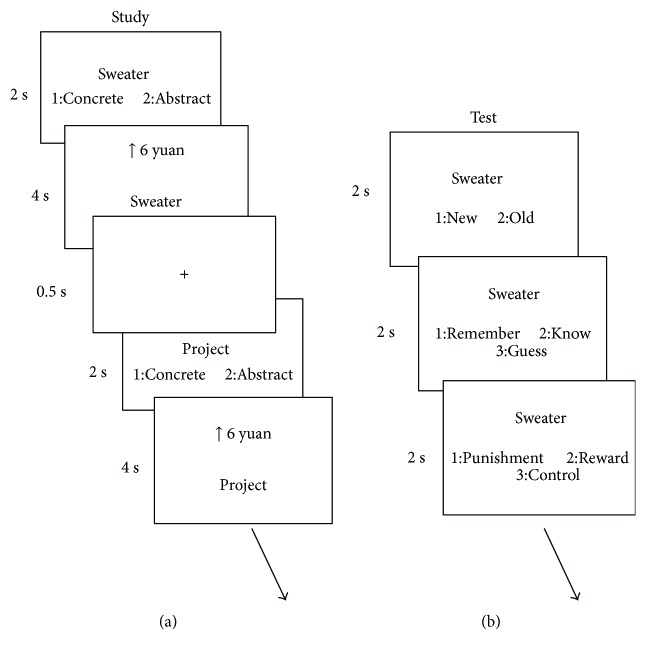
Procedure of the study and test sessions in experiment 2. During encoding, participants first performed a concreteness judgment for each word and then tried to remember the word with different motivational cues. During retrieval, the participants made word recognition, R/K/G judgment, and source judgment for each word. Chinese words are replaced by English words for illustration purpose.

During the encoding phase, the participants were told to memorize the words in different motivational conditions. In each trial, participants were first presented a neutral word for 2 s, during which they made a concrete/abstract judgment with the word (Figure 3(a)). Then the word was presented again for 4 s with the motivational cues. Three kinds of symbols represented motivational cues, “0 yuan,” “↓ 6 yuan,” and “↑ 6 yuan.” The “↑ 6 yuan” meant that the participants would get the reward of 6 yuan for each word if the word was correctly recognized in the recognition task; “↓ 6 yuan” meant that the participants would get a deduction of 6 yuan for each word if the word was not correctly recognized in the recognition task; “0 yuan” meant that there was neither reward nor punishment regardless of whether the participants recognized the word in the recognition task. The participants were asked to remember the association of word and the motivational condition. At the end of the learning phase, they were asked to fill in a motivational intensity scale, using numbers 1 to 5 to evaluate the motivational intensity of memorizing words subjectively in different incentive conditions.

During the test phase, in each trial, the participants performed three tasks: an old/new recognition test, a remember/know/guess (R/K/G) judgment, and a motivational condition (reward/control/punishment) judgment (Figure 3(b)). The procedure was the same as experiment 1, except that in the source memory task, the participants were asked to judge the motivational condition the word was associated with. The words were presented in a pseudorandom order, so that no more than three words from the same incentive condition or old/new condition were presented continuously.

To control for the response bias, two steps were further applied. First, before the experiment, the participants were informed that the amount of reward and punishment was independent of the test pay. Second, before the test phase, the participants were told that the judgment of new words would be rewarded and punished. When a new word was judged as new, the participants would be given a 2-yuan reward; when a new word was judged as old, the participants would get a deduction of 2-yuan punishment.

Before the formal test, the participants had an exercise with feedback of their performance, and they saw their own rewards and punishments at the end of the exercise. In the formal test phase, no feedback was provided.

After the study, the participants were asked to fill in the questionnaire about the motivational intensity (range: 1–5): How strong is the motivation to avoid losing money when you see the cue of “↓ 6 yuan”? How strong is the motivation to gain money when you see the cue of “↑ 6 yuan”? How strong is the motivation to gain more money when you see the cue of “0 yuan”?

The participants learned the 240 words in the same day and then performed the recognition tests at four time intervals. The material varied for different time intervals. Before each test phase, to avoid a rehearsal from the study phase, the participants were asked to count backward by 7 continuously from 1000 for 5 minutes. In addition, to prevent the participants from rehearsing the stimuli after the study phase, they were reminded that it was not necessary to retrieve or forget the stimuli intentionally.

#### 2.2.4. Data Analysis

The analysis was the same as that in experiment 1. The Hit rate, FA rate, corrected recognition (Hit-FA), and mean RTs were calculated and analyzed separately using a repeated measures ANOVA with the time interval (10 min, 1 day, 1 week, and 1 month) and the motivational context (reward, punishment, and control) as within-subject factors. Two subjects' data were excluded due to lower hit rates (>2 SD). The remaining 26 subjects' results were used for data analysis.

## 3. Results

### 3.1. Experiment 1

During the encoding task, the participants rated the sentence vividly with an average score of 3.14 ± 0.53. The ANOVA results showed that there was no significant difference in vividness for different contexts (positive: 3.20 ± 0.51; negative: 3.12 ± 0.55; neutral: 3.12 ± 0.58. *P* = 0.81). This ensured that stimuli in each condition were efficiently encoded.

**Figure 4:**
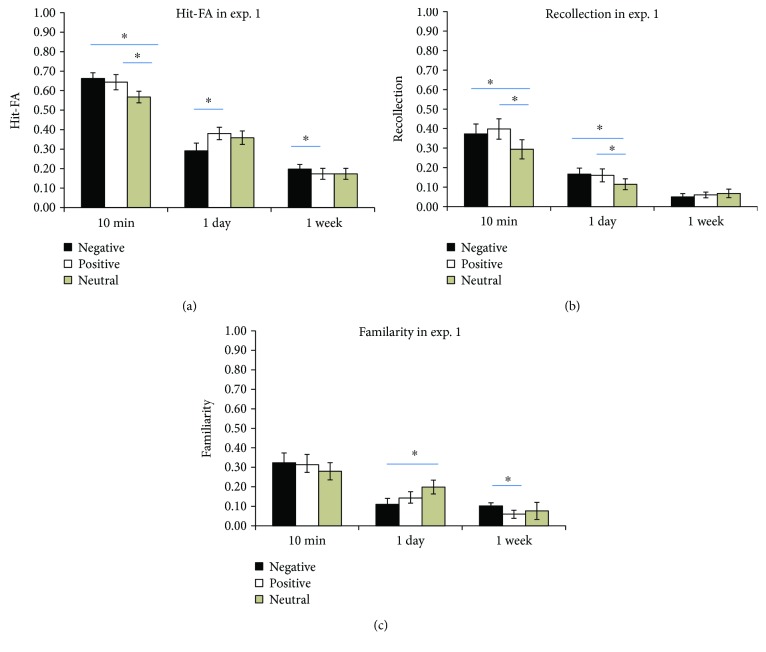
Results of experiment 1. Corrected recognition (a). Contribution of recollection (b) and familiarity (c). The error bars represent the standard errors of the means. ∗ represent *P* < 0.05.

For the corrected recognition, the results showed that memory accuracy decreased over time (*F*(2,46) = 130.52, *P* < 0.001, *η*^2^ = 0.85) (Figure 4). There was a significant interaction between retention interval and context (*F*(4,92) = 5.30, *P* = 0.001, *η*^2^ = 0.19). Further analysis showed that there was higher corrected recognition for positive (*P* = 0.007) and negative (*P* = 0.003) conditions than for the neutral condition at 10 min and higher for the positive than for the negative condition at 1 day (*P* = 0.05). There was higher corrected recognition for the negative than for the positive condition (*P* = 0.035) at 1 week (Figure 4(a)). In addition, for memory in negative contexts, recognition performance decreased from 10 min to 1 day (*P* < 0.001) and stayed stable from 1 day to 1 week (*P* = 0.149). For memory in positive and neutral contexts, recognition performance decreased from 10 min to 1 day (*P* < 0.001) and from 1 day to 1 week (*P*′s < 0.005). The corrected recognition in different conditions was significantly higher than expected by chance (0) (*P*′s < 0.05). For the RTs, there was a significant effect for the time interval (*F*(2,46) = 5.21, *P* = 0.01, *η*^2^ = 0.207) but no significant effect of context or the interaction between context and interval (*P*′s > 0.05).

The Hit rate decreased over time (*F*(2,46) = 57.11, *P* < 0.001, *η*^2^ = 0.713). The effect of interaction was significant (*F*(4, 92) = 4.05, *P* = 0.005, *η*^2^ = 0.15). Memories for both negative and positive contexts were higher than those for the neutral context at 10 min interval (*P*′s < 0.01), and the memory for positive and negative contexts did not differ (*P* = 0.99). There were no significant differences among contexts at 1-day and 1-week intervals (*P*′s > 0.10). For the FA rate, there was significant effect of time interval (*F*(2,46) = 13.28, *P* < 0.001, *η*^2^ = 0.37), increasing from 10 min to 1 day (*P* < 0.01) and from 1 day to 1 week (*P* < 0.01), but the interaction and context effect were not significant (*P*′s > 0.20).

Regarding the contribution of recollection, there was a significant effect of time interval (*F*(2, 46) = 40.89, *P* < 0.001, *η*^2^ = 0.64), showing that the recollection estimates decreased over time. The interaction between time interval and context was significant (*F*(4, 92) = 3.87, *P* = 0.011, *η*^2^ = 0.14). Further analysis showed a higher contribution of recollection for the positive (*P* = 0.001) and the negative (*P* = 0.008) than for the neutral at 10 min. There was a higher contribution of recollection for negative than for neutral at 1 day (*P* = 0.022) (Figure 4(b)). The recollection estimates in different conditions were significantly higher than expected by chance (0) (*P*′s < 0.02).

Regarding the contribution of familiarity, there was a significant effect of time interval (*F*(2,46) = 23.58, *P* < 0.001, *η*^2^ = 0.506). The interaction between time interval and context was significant (*F*(4,92) = 5.506, *P* = 0.002, *η*^2^ = 0.193). Further analysis showed that there was a higher contribution of familiarity for the negative than for the positive (*P* = 0.019) at 1 week (Figure 4(c)). In addition, the contribution of familiarity decreased from 10 min to 1 week for the positive condition (*P*′s < 0.01) and decreased from 10 min to 1 day for the negative condition (*P* < 0.001). The familiarity estimates in different conditions were significantly higher than expected by chance (0) (*P*′s < 0.05).

We also included the process (recollection, familiarity) as a factor in the ANOVA analysis. The results showed a significant interaction among process, time, and context (*F*(4,84) = 3.51, *P* < 0.01, *η*^2^ = 0.14). The effect of process was not significant (*F* < 1). Further analysis showed that there was a higher contribution of familiarity than of recollection for the negative context at 1 week (*P* = 0.006). It suggested that memory performance relies on both recollection and familiarity, and the higher memory for the negative context at 1 week is associated with the familiarity contribution.

Regarding the memory for the emotion/neutral judgment, the results showed that memory accuracy decreased over time (*F*(2,46) = 17.60, *P* < 0.001, *η*^2^ = 0.434). There was a significant main effect of emotion (*F*(1,23) = 11.69, *P* = 0.002, *η*^2^ = 0.337), showing a higher accuracy for the neutral than for the emotional (*P* = 0.002). Only the source memory for the neutral contexts was above the chance level. Regarding the memory for the negative/positive judgment, the results showed that memory accuracy decreased over time (*F*(2,46) = 3.4, *P* = 0.047, *η*^2^ = 0.129). There was no significant main effect of emotion (*F*(1,23) = 1.04, *P* = 0.318, *η*^2^ = 0.043), showing no difference of accuracy between negative and positive source memory. The source memory was not above chance level in the negative/positive source judgment. We also calculated the source memory out of the corrected trials, but the result remained the same. It suggested that subjects could not remember the context conditions the words had during encoding.

In sum, the main result of experiment 1 was that there was significant interaction between time and context for corrected recognition. The memory with negative and positive contexts changed over time in different patterns. The positive advantage (versus negative) occurred at the 1-day interval, and negative advantage (versus positive) occurred at the 1-week interval. In addition, the positive advantage at 1 day was driven by both recollection and familiarity processes, whereas the negative advantage at 1 week was driven by the familiarity process. The memory for contextual information was not above chance level; therefore, it was not enhanced.

### 3.2. Experiment 2

The subjective rating scores after the study showed that the scores of motivation were higher for both reward (4.07 ± 0.71) and punishment (4.24 ± 0.64) conditions than the control (3.07 ± 1.07) condition, *F*(2,56) = 21.367, *P* < 0.001, but there was no significant difference between the reward and the punishment conditions (*P* = 0.80). The post hoc reports ensured that the motivational levels of reward and punishment were matched.

**Figure 5:**
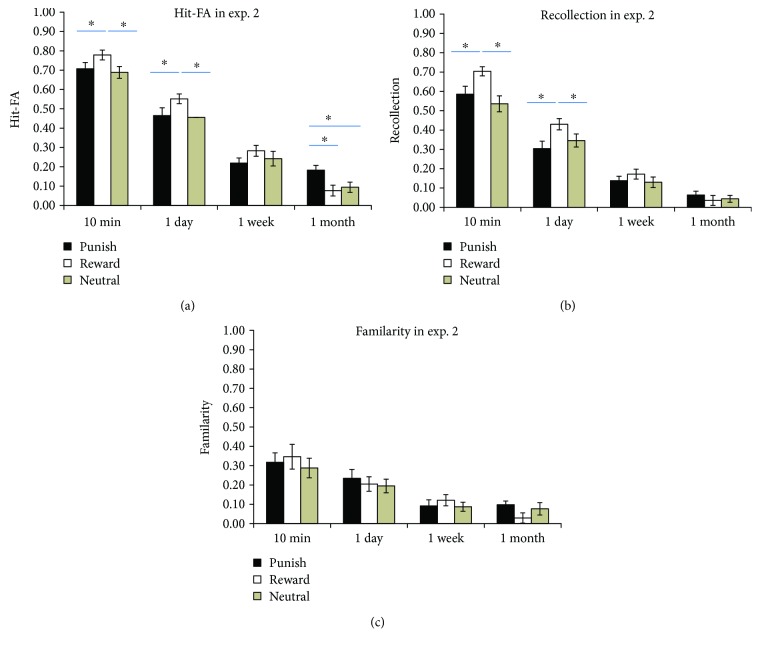
Results of experiment 2. Corrected recognition (a). Contribution of recollection (b) and familiarity (c). The error bars represent the standard errors of the means. ∗ represent *P* < 0.05.

For the corrected recognition, the results showed that memory accuracy decreased over time (*F*(3,75) = 196.71, *P* < 0.001, *η*^2^ = 0.887) (Figure 5(a)). There was a significant main effect of motivation (*F*(2,50) = 4.72, *P* = 0.013, *η*^2^ = 0.159), showing that memory for the reward condition was higher than that for the control condition (*P* = 0.022), but the memory for the punishment condition was similar to that of the control condition (*P* = 0.53). There was a significant interaction between time interval and context (*F*(6,150) = 4.07, *P* = 0.001, *η*^2^ = 0.14). Further analysis showed higher corrected recognition for the reward condition than for the punishment (*P* = 0.012) and control conditions at 10 min (*P*′s < 0.01) and 1 day (*P*′s < 0.05), but there was higher corrected recognition for the punishment than for the reward (*P* = 0.007) and control (*P* = 0.026) at the 1-month interval, with no significant difference between the reward and control condition (*P* > 0.05). It suggested that memory for the punishment condition is forgotten more slowly than the other two conditions. The corrected recognition in different conditions was significantly higher than expected by chance (0) (*P*′s < 0.05). For the RTs, there were no significant effects of time interval, context, or their interaction (*P*′s > 0.05).

Similar to the corrected recognition, the Hit rate decreased over time (*F*(3,75) = 70.36, *P* < 0.001, *η*^2^ = 0.74). There was a significant main effect of motivational condition (*F*(2,50) = 4.85, *P* = 0.013, *η*^2^ = 0.162), showing that memory performance for the reward condition was higher than that for the punishment and control conditions (*P*′s < 0.02), but the memory for the punishment and control conditions did not differ (*P* = 0.10). There was no significant interaction between context and time interval (*F*(6,150) = 1.56, *P* = 0.18, *η*^2^ = 0.06). For the FA rate, there was a significant effect of time interval (*F*(3,75) = 56.68, *P* < 0.001, *η*^2^ = 0.69), increasing from 10 min to 1 day (*P* < 0.001) and from 1 day to 1 week (*P* < 0.001), and remained stable from 1 week to 1 month (*P* > 0.05). The interaction was significant (*F*(6,150) = 2.90, *P* = 0.01, *η*^2^ = 0.104). Further analysis showed that the FA rate was higher for the punishment (versus reward) condition at 10 min (*P* = 0.06), but the opposite at the 1-week interval (*P* = 0.008).

Regarding the contribution of recollection, there was a significant effect of time interval (*F*(3,75) = 140, *P* < 0.001, *η*^2^ = 0.848). The interaction between context and time interval was significant (*F*(6,150) = 5, *P* = 0.001, *η*^2^ = 0.167). Further analysis showed that there was a higher contribution of recollection for the reward condition than for the punishment and control conditions at 10 min (*P* < 0.001) and 1 day (*P* < 0.05) (Figure 5(b)). The estimates in different conditions were significantly higher than expected by chance (0) (*P*′s < 0.05) except for that in the reward condition at the 1-month interval (*P* = 0.17). It suggested that reward advantage is attributed to the recollection process, but recollection decreased quickly in the reward condition. The contribution of familiarity decreased over time (*F*(3,75) = 24.61, *P* < 0.001, *η*^2^ = 0.50). The interaction of interval and context was not significant (*F* < 1, *P* > 0.60) (Figure 5(c)). The estimates in different conditions were significantly higher than the expected by chance (0) (*P*′s < 0.05) except for that in the reward condition at the 1-month interval (*P* = 0.11).

We also included the process (recollection, familiarity) as a factor in the ANOVA analysis. The results showed a significant effect of process (*F*(1,24) = 14.91, *P* = 0.001, *η*^2^ = 0.38). There was a marginally significant interaction among process, time, and context (*F*(6,144) = 1.87, *P* = 0.08, *η*^2^ = 0.07). Further analysis showed that there was a higher contribution of recollection than familiarity in the positive and neutral contexts at 10 min and 1 day (*P*′s < 0.01) and in the negative context only at 10 min (*P* = 0.001). It suggested that memory performance relies on recollection at shorter intervals, but relies on both processes at longer intervals. The higher memory for the negative context at 1 week might be associated with both recollection and familiarity contributions.

The source memory was not above chance level (0.33) from 1 day to 1 month (*P*′s > 0.05). At the 10 min interval, there was higher accuracy for the reward than for the punishment condition (*P* = 0.04), but both conditions did not significantly differ from the control condition (*P*′s > 0.30). There was no significant effect of interaction (*F*(6,150) = 2.05, *P* = 0.081, *η*^2^ = 0.08). The result remained the same when corrected values of source memory were used for analysis.

In sum, similar to those in experiment 1, there was significant interaction between time and context for corrected recognition in experiment 2. The memory by reward decreased quickly and depended on the recollection process, whereas the memory by punishment contexts decreased slower after 1 week. The results of experiment 2 suggested that memory by punishment and reward motivation produces different rates of forgetting.

## 4. Discussion

In this study, the factors of type of context and time interval were manipulated to explore their effects on memory for neutral targets. We asked whether these factors influenced memory after different emotional contexts and motivational conditions over time. By controlling for the arousal level and motivational intensity, the results showed significant interaction between context and time interval in experiments 1 and 2. Compared to negative and punishment conditions, words in positive and reward contexts were recognized better at shorter intervals, which was associated with recollection process. In contrast, the words in negative and punishment contexts were recognized better at longer intervals, which was mainly associated with familiarity process. The results clarified how contextual and motivational cues influence memory at both short and long intervals and highlighted the contextual feature in memory formation and retention.

### 4.1. Positive versus Negative Contexts from 10 Min to 1 Week

One novelty of the study was that we compared the memory performance in the positive and negative contexts in different time intervals and found that they had different effects on memory of neutral targets over time. Specifically, memory in the positive contexts was forgotten from 10 min to 1 week, whereas memory in the negative contexts was forgotten only from 10 min to 1 day. Thus, the enhanced memory in the positive context was obvious at shorter intervals, which relied on the recollection, whereas the negative (versus positive) enhancement was obvious at longer intervals, which mainly relied on the familiarity process.

The results supported our hypothesis that memory enhancement in the positive and negative contexts is different and time-dependent. The reason for the time-dependent memory enhancement is associated with the underlying processes. First, positive and negative emotions have different characteristics, and memories associated with them rely differentially on recollection and familiarity processes. Compared with negative emotion, positive stimuli broaden the scope of attention [35], which results in increased perceptual processing of task-irrelevant information [15]. The memory for the neutral words may thus have more associated contextual information, leading to increased recollection contribution [36]. In contrast, negative contexts reduced memory specificity and lead to overgeneralized emotional memory [37]. The enhancement after shock as contexts depended on item familiarity but not recollection [9].

Second, there are different forgetting rates for memories depended on recollection and familiarity [25, 33]. Memory relying on the recollection process is subjected to decay, whereas that relying on familiarity process is more resistant to decay over time [25]. In experiment 1, the interactions between time interval and context were significant for both recollection and familiarity processes. There was higher recollection contribution for the positive and negative contexts than the neutral at 10 min and higher familiarity for the negative than for the positive at 1 week. The contribution of familiarity decreased more slowly for the negative than the positive contexts. This explains why memory in positive contexts was more enhanced at 10 min and 1-day intervals, and memory in negative contexts could remain at 1 week. Cairney et al. also showed that memory of target pictures in negative contexts was less likely to be lost [38]. The results provided evidences that the positive and negative contexts are associated different processes to enhance memory with the passage of time.

### 4.2. Reward versus Punishment/Stressful Contexts from 10 Min to 1 Month

Previous studies have suggested that the effect of motivational contexts occurs right after the encoding [23], 24 hours later [5], and even three weeks later [6]. The current study clarified the extent the reward and punishment contexts modulated memory at different time intervals. Neutral words in reward contexts were recognized at higher level than those in the punishment and neutral contexts at 10 min and 1 day, and the words in the punishment contexts were recognized better than those in the positive and neutral contexts at 1 month. Thus, words in the stress/punishment context were forgotten more slowly than those presented in the reward contexts.

In this study, we found the reward-related enhancement occurred at both 10 min and 1 day. The memory enhancement was also observed when the test delay was immediately or minutes after encoding [13, 23, 39]. Reward but not punishment context enhanced the memory when the test was performed minutes after encoding [8, 34]. Although Shigemune et al. found comparable enhancement for both reward and punishment contexts immediately after encoding [23], they measured item with source memory correct rather than item memory.

Some studies only found the reward-related enhancement at 24 h but not immediately after study [20, 21]. The results of reward-related enhancement at both 10 min and 1 day in this study are not contradictory to the retroactive enhancement, because we presented the motivational contexts with the neutral words for 4 s, rather than after the neutral words [20, 21]. The mechanisms for proactive or retroactive enhancement are different. When contexts are presented before or right with the stimuli, both encoding and consolidation processes are possible ways to enhance proactive memory [13, 23, 39, 40]. In contrast, the retroactive memory emphasizes that the reward and punishment contexts modulate memory consolidation [20–22]. For example, if neutral objects were paired with shock, the retroactive memory for the objects in the same category was selectively enhanced 6 h and 24 h later [22]. Similar pattern occurred when the category was rewarded [39, 41, 42]. Also note that in Dunsmoor et al. [22], in addition to the retroactive memory enhancement, they found the memory enhancement for the stimuli that were paired with shocks immediately after encoding, 6 h and 24 h later, which was consistent with our findings.

Similar to that for emotional contexts, the reason for the time-dependent memory enhancement for motivational contexts is associated with the underlying processes. The results showed that the enhancement for the reward contexts at short intervals was associated with recollection contribution, and that for the punishment context at longer interval was associated with both recollection and familiarity contribution. It is suggested that under the reward condition, subjects are more likely to remember the details of the neutral stimuli [6]. There was a higher contribution of recollection than familiarity in the positive and neutral contexts at 10 min and 1-day intervals. Previous studies have also shown that reward improved memory by selectively enhancing recollection process rather than familiarity [12, 13, 43]. As the recollection process is subjected to decay over time [25, 33], the memory enhancement for the reward contexts diminished over time. By contrast, punishment motivation facilitates global representation of context [8], so the memory under the punishment is more schematic. At the 1-month interval, the recollection and familiarity estimates were both above chance level for the punishment condition but not for the reward condition. Thus, the different forgetting pattern for the reward and punishment context reflects that the underlying processes support memory representations.

### 4.3. Memory for Contextual Information

Different from the enhanced memory for the target words, we did not find significant memory enhancement for contextual information. The source memory did not exceed the chance level in most conditions in experiments 1 and 2.

Our results suggested that memory enhancement does not apply to all information related to the targets [11, 13, 44, 45]. The memory of contextual information may be automatic and implicit. Different from remembering the emotional target, the relation between the target and context is sparse. Emotion and motivation selectively affect the recollection of target items rather than the contexts [46, 47] and do not enhance the memory of extrinsic or contextual information [46]. In addition, even participants were more confident that they remembered pictures, the performance was irrespective of actual encoded context [45]. Thus, it is the subjective feeling of the context, not the objective context that determines the memory for contextual information.

On the other hand, even the source memory is implicit, the context could influence the subsequent cognitive processes, such as decision making and valence judgment [37, 48]. These results suggested that emotional and motivational contexts influence memory retrieval in the absence of overt behavioral differences [49]. Participants may reexperience the emotion automatically during retrieval of neutral targets [49, 50]. Also, note that the source memory may be related to source type. For example, Shigemune et al. tested the position of the word, rather than the contextual condition, and found higher source memory for emotional conditions [23]. Further studies could use implicit memory tasks or other sources to clarify whether the source information is retained.

### 4.4. Relationships between Emotional and Motivational Contexts

Although emotional pictures and monetary cues were different in various aspects, our results showed that both of them enhanced the memory for the neutral targets. In addition, the memory enhancement in experiments 1 and 2 had similar characteristics. For example, the positive and reward contexts enhanced memory at shorter time intervals, which were driven by the recollection process; whereas, the negative and punishment contexts enhanced memory at longer intervals, which were mainly driven by the familiarity process. In addition, the memory for contextual information was low.

There is a close relationship between emotion and motivation in both behavior and brain activation. For example, in a study by Delgado et al., mild shock and loss of money were used as aversive unconditioned stimuli separately and were paired with one of two conditioned stimuli [26]. The results showed that the striatum was involved in both shock and money conditions. In addition, positive stimuli enhance reward-related memory performance and activation in the midbrain [27, 51]. Arousal level interacted with the motivational condition to influence subsequent memory performance [34]. These results suggested that positive and reward and negative and punishment may interact and share similar brain mechanisms, including the midbrain reward system and the amygdala. The results in our study provided behavioral evidences that the positive and reward and negative and punishment context had similar mechanisms in enhancing memory of the neutral targets. Both types of the contexts could modulate the medial temporal memory system, making the behavioral consequences similar.

On the other hand, the effects of the two types of contexts differed in several aspects. In this study, although we are unable to compare the two types of contexts directly due to various experimental manipulations, some differences may be inferred from our results. First, memory performance in the motivational contexts was higher than that in the emotional contexts, especially at shorter intervals. The memory enhancement for the motivational contexts depended more on recollection, because the results showed higher recollection estimates than familiarity in experiment 2, but the two processes were comparable in experiment 1. Second, the memory in the emotional contexts seemed to be forgotten more quickly than that in the motivational contexts. Particularly, memory enhancement for the negative contexts occurred at 1 week, whereas that for the punishment contexts at 1 month. We call for caution in interpreting this result and advise further studies to clarify whether the memory in the motivational contexts lasts longer than that in the emotional contexts. The difference in memory performance may be a possible source of divergent results. Third, the memory for motivational contexts was above chance level at the 10 min interval, but the memory for emotional contexts was not above chance level from 10 min to 1 week. These results suggested that reward and punishment contexts might facilitate memory details of both target item and source information at shorter intervals. How these differences happen at the level of neural activation needs future investigations.

### 4.5. Conclusions

In summary, the results of this study clarified the cognitive mechanisms of how contextual and motivational cues influence memory formation and consolidation. The positive and reward contexts enhanced memory by recollection process and lasted for a shorter time. The negative and punishment contexts enhanced memory mainly be familiarity process and lasted at longer intervals. The results provided evidence that emotional and motivational cues influence memory processes in different dimensions and highlight that different processes mediate memory enhancement in different contexts.

The current findings have significant implications for practice. It is clear that different contexts had impacts on recent and remote memories that were associated with these contexts. On the one hand, we are exposed to a large amount of information, and most of it is devoid of emotional and motivational values. To enhance memory, one possible way is to combine emotional or motivational contexts with the information. Moreover, because these contexts differ in their effects on retention time and detail or gist part of memory, different contexts could be chosen to enhance memory. On the other hand, it sheds light on how stress induces long-term consequences in memory and many mental disorders [52, 53]. For example, acute stress could be induced by negative events, so the results are similar to those in negative and punishment contexts [4]. But as stress level differs in its intensity and duration, the effects of stress on memory are complicated and need careful manipulation and interpretations.
